# The effects of auditory stimulation on heart rate variability in healthy individuals with normal hearing and with hearing loss: a systematic review and meta-analysis

**DOI:** 10.1590/2317-1782/20242023111en

**Published:** 2024-05-31

**Authors:** Bárbara Cristiane Sordi Silva, Eliene Silva Araújo, Vitor Engrácia Valenti, Lilian Cássia Bórnia Jacob, Katia de Freitas Alvarenga

**Affiliations:** 1 Departamento de Fonoaudiologia, Faculdade de Odontologia de Bauru – FOB, Universidade de São Paulo – USP - Bauru (SP), Brasil.; 2 Departamento de Fonoaudiologia, Universidade Federal do Rio Grande do Norte – UFRN - Natal (RN), Brasil.; 3 Departamento de Fonoaudiologia, Faculdade de Filosofia e Ciências, Universidade Estadual Paulista – UNESP - Marília (SP), Brasil.

**Keywords:** Autonomic, Nervous System, Acoustic Stimulation, Hearing, Physiology, Systematic Review

## Abstract

**Purpose:**

To analyze the effects of auditory stimulation on heart rate variability (HRV) indices in healthy individuals with normal hearing and with hearing loss, regardless of type and/or grade, by means of a systematic review.

**Research strategies:**

This is a systematic review with a meta-analysis that addresses the following question: in healthy individuals with normal hearing and/or with hearing loss, what are the effects of auditory stimulation on HRV indices in comparison to silence? We consulted the Cochrane Library, Embase, LILACS, PubMed, Web of Science, and Scopus databases and the gray literature (Google Scholar, OpenGrey, and ProQuest).

**Selection criteria:**

There were no restrictions as to period or language of publication.

**Data analysis:**

We identified 451 records, an additional 261 in the gray literature, and five studies in a search through the references, resulting in a total of 717 records, with 171 duplicate records. After screening the titles and abstracts of 546 studies, we excluded 490 and considered 56 studies in full to assess their eligibility.

**Results:**

Nine of these studies were included in the systematic review, eight of which were suitable for the meta-analysis.

**Conclusion:**

It is suggested that auditory stimulation may influence the RMSSD, pNN50, SDNN, RRTri and SD2 indices of HRV in healthy adults with normal hearing.

## INTRODUCTION

Previous research, carried out in the 1970s/80s, analyzed the clinical utility of the heart rate response to assess hearing in children and came up with favorable findings at the time, encouraging further research^(1-3)^, despite the paucity of more recent studies with this purpose.

From another perspective, currently, studies in the area have focused mainly on analyzing the association between Heart Rate Variability (HRV) and auditory evoked potentials, in adults with normal hearing, reporting the interaction between the autonomic control of heart rate with the cochlear nerve^(4)^, between the heart rhythm with the thalamo-cortical, cortical-cortical and auditory cortex^(5)^, and between cardiac autonomic modulation with the Cortical Auditory Evoked Potential^(6)^. Other research analyzing HRV, in individuals without and with hearing loss, through tasks for measuring listening effort^(7-9)^

Therefore, when considering the scientific evidence documented by literature that demonstrated the relationship between heart rhythm control and hearing, we hypothesized about the possibility that there is an association between HRV—the oscillations in the time intervals between consecutive heartbeats—and auditory sensitivity.

Thus, the relationship between heart rate and hearing is discussed in the literature, but there is no reviews were found with a specific focus on surveying the association between HRV and auditory sensitivity. In this way, a systematic review is justified with a comprehensive search strategy on the subject, with the aim to analyze the effects of auditory stimulation on HRV indices in healthy individuals with normal hearing and with hearing loss, regardless of type and/or grade.

## RESEARCH STRATEGIES

### Protocol and registration

The systematic review was developed in accordance with the Preferred Reporting Items for Systematic Reviews and Meta-Analyses (PRISMA)^(10)^, and its r protocol was registered in the International Prospective Register of Systematic Reviews (PROSPERO)^(11)^ website – CRD42021192659^(12)^. The Population, Intervention, Comparator, and Outcome (PICO) acronym was used to establish the eligibility criteria for the research question: in healthy individuals with normal hearing and/or with hearing loss, what are the effects of auditory stimulation on HRV indices in comparison to the silence?

### Information sources and search strategy

Appropriate word combinations were adapted to six electronic databases selected as information sources: Cochrane Library, Embase, Latin American and Caribbean Literature in Health Sciences (LILACS), PubMed/Medline, Web of Science, and Scopus. In addition, gray literature was used as a source of information through Google Scholar, OpenGrey, and ProQuest Dissertation and Thesis (Appendix A). A manual search of references was carried out in all included studies. An expert on the subject was consulted to verify suggestions of references relevant articles that could be included. There were no restrictions as to period or language of publication. Database searches were performed on November 15, 2021 and updated on November 10, 2022 and Endnote® software was used to manage and remove duplicate references^(13)^.

## SELECTION CRITERIA

### Eligibility criteria

To consider the eligibility of studies to be included/excluded from this review, the acronym “PICOs” was used:

- Population (P): We considered healthy individuals of both sexes of any age with normal hearing and/or with unilateral or bilateral hearing loss of all types or degrees. We excluded studies on individuals with any disorders and/or health conditions other than hearing loss and on individuals using medication that could influence control over heart rhythm;
- Intervention (I): We considered auditory stimuli presented by air conduction, regardless of type, duration, intensity, and calibration unit, simultaneously with the evaluation of the HRV indices. To avoid possible interference, we excluded studies with multisensory stimulation and/or those which executed auditory stimuli and concomitant tasks;
- Comparison (C): We considered comparisons to the absence of auditory stimuli (silence and at rest) prior to the intervention;
- Outcomes (O): We analyzed the simultaneous effects of auditory stimulation on HRV indices. Studies that assessed HRV immediately or long after auditory stimulation were excluded. We observed primary outcomes: time domain—RMSSD index, frequency domain—HF index (n.u.), and geometric analysis—SD1 index. We also took note of secondary outcomes, namely other HRV indices presented in the included studies;
- Study design(s): We considered randomized clinical studies or non-randomized, cross-sectional observational studies, and cohort or case-control^(14)^.

### Selection process

The selection of articles was carried out in two phases. Prior to beginning the selection process, a calibration was performed between the reviewers. In the phase 1, two reviewers independently reviewed the titles and abstracts of all references through the Rayyan — a web and mobile app for systematic reviews^(15)^, blinding reviewers, which resulted in almost perfect agreement, with Kappa Coefficient = 0.98^(16)^. A third reviewer was consulted when disagreements arose. All papers that did not meet the eligibility criteria previously established were excluded at this stage. In the phase 2, the full text of the articles selected in the first phase were read.

## DATA ANALYSIS

### Data collection process and data items

Two reviewers collected data of interest from the included studies. The collected data consisted of (i) characteristics of the study (author, year of publication, country and study design); (ii) characteristics of the population (sample size, age, sex, clinical health history, and audiological data); (iii) characteristics of the intervention (type, intensity, and duration of the auditory stimulus and calibration unit and transducer used); and (iv) characteristics of the outcome relative to the HRV assessment (equipment and parameters used, duration of HRV measurements and the indices measured, and quantitative results with numerical variables, including n sample size, mean, standard deviation or confidence interval, and p-value).

In the presence of incomplete or missing data in the article, two attempts were made to contact the corresponding authors identified in the articles, with an interval of two weeks. When it was impossible to obtain information, either due to the absence of responses or unavailable data, we performed only a descriptive analysis of the results or the study was excluded.

### Study risk of bias assessment

Two reviewers assessed the methodological quality and risk of bias (Kappa coefficient = 0.97) of the included studies by using the JBI Critical Appraisal tool^(17)^. The checklist was selected according to the included study design. The questions included four response options: “yes,” “no,” “uncertain,” and “not applicable.” The risk of bias percentage for each study was determined by the occurrence of the answer “yes,” while the answer “not applicable” was not factored into the calculation. The classifications for risk of bias were as follows: high (≤ 49% for “yes” score), moderate (50-69% for “yes” score), and low (≥ 70% for “yes” score). Disagreements were resolved through discussion and, in the presence of a lack of consensus, a third reviewer was involved. The Review Manager 5.4® software was used to generate figures.

### Effect measures

The primary and secondary outcomes were summarized in effect measures. Since these are continuous data, the difference between means (MD) was calculated by comparing baseline values for each outcome with their values during the intervention.

To evaluate the effects of auditory stimulation on HRV indices, we pooled the data for meta-analysis. We used Review Manager version 5.4® to perform the statistical evaluation and calculated the differences in means by using the number of individuals and the mean/standard deviation for the control (absence of auditory stimulus) and intervention (presence of auditory stimulus) arms in the inverse-variance statistical method, with a random effects model and a 95% confidence interval. We considered all the data for meta-analysis regardless of the auditory stimulus, intensity, or duration of HRV measurements; as a result, some studies were included more than once in the statistical analysis, depending on the methodology of each one. In cases where it was necessary to enter repeated values, we determined the proportional distribution of the n sample size^(18)^. The study by Roque et al.^(19)^ divided the participants into two groups, which were analyzed separately.

Statistical heterogeneity was quantified by Higgins’ inconsistency test (I^2^), which was interpreted as follows: 0-40% = may not be important, 30-60% = moderate heterogeneity, 50-90% = substantial heterogeneity, and 75-100% = considerable heterogeneity^(20,21)^. We also applied the tau-squared and chi-squared tests^(21)^.

### Subgroup analysis (primary outcomes)

If statistical heterogeneity were found, we would conducted an analysis of subgroups to explore possible confounding factors for the analysis: (i) t influence of sex; (ii) influence of the type of auditory stimulus; (iii) influence of the intensity of the auditory stimulus; and (iv) influence of the duration of HRV measurements in the presence of the auditory stimulus.

### Reporting on bias assessment

We intended to analyze publication bias by using funnel plots to estimate intervention effect through the standard error; however, this assessment was not possible as fewer than 10 studies were included in the meta-analysis performed for each outcome, preventing the funnel plot asymmetry test^(20,21)^. In addition, to reduce the probability of occurrence of a publication bias, a broad search strategy in databases and gray literature, were carried out.

### Certainty assessment

The level of certainty of evidence was assessed by the Grading of Recommendations Assessment, Development, and Evaluations (GRADE) tool, with four levels of classification: very low, low, moderate, and high, according to the level of certainty judged according to the following aspects: risk of bias, inconsistency, indirect evidence, imprecision, and publication bias. Two evaluators used the GRADEpro online platform^(22)^, Kappa coefficient = 1.00.

## RESULTS

### Study Selection

**Figure 1:**
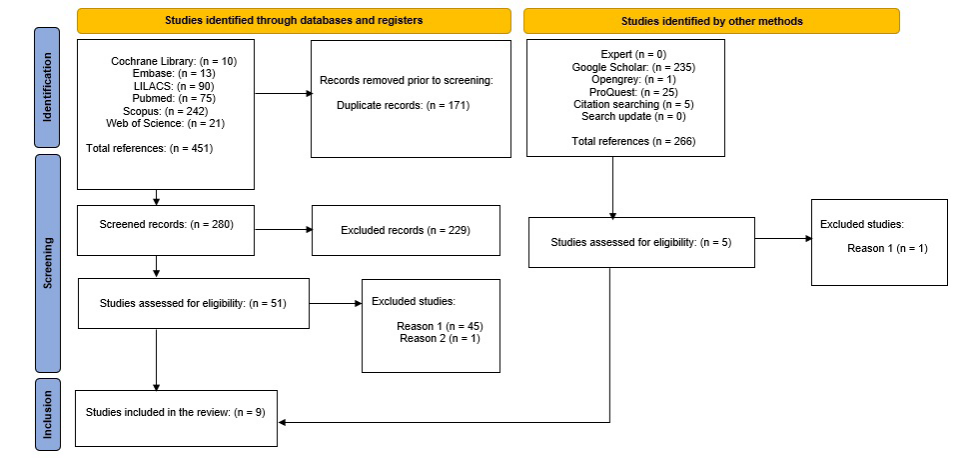
Flow diagram of the literature search and the selection criteria.

We identified a total of 451 records, an additional 261 records in the gray literature, and five studies in our search through the references, resulting in a total of 717 records, including 171 duplicate records. Next, we screened the titles and abstracts of 546 studies and excluded 490 (phase 1), which left 56 studies to consider in their entirety for eligibility (phase 2), of which 47 were excluded (Appendix B) and, nine articles were included (Figure 1), eight (88.89%) of which were suitable for the meta-analysis^(19,23-29)^. No additional studies were included from the consultation with experts.

Caption: Reason 1 = exclusion due to intervention; Reason 2 = exclusion due to outcome.

Source: Adapted from by Page, M. J. et. al. The PRISMA 2020 statement: an updated guideline for reporting systematic reviews. BMJ 2021;372:n71

### Study Characteristics

All the included studies were cross-sectional observational studies conducted among healthy adults without hearing loss that were published in English between the years 2010 and 2016. Eight (88.9%) were conducted in Brazil^(19,23-29)^, and one (11.1%) was conducted in Taiwan^(30)^. Various types of auditory stimuli with varying durations and intensities were employed. HRV was assessed in 187 individuals of both sexes with a mean age of 22.5±2.1 years in the absence of auditory stimuli—in silence and at rest—and in the presence of auditory stimuli. The Tables 1 and 2 synthesize the individual characteristics of the population, intervention, comparison, and outcomes of the included studies.

**Table 1:** Synthesis of the individual descriptive characteristics of the population of the included studies

**Author (year)**	**Population**
Lee et al.^(30)^	16 individuals, 6 males and 10 females, mean age 25.9±6.4 years, with normal hearing and no medical history of hypertension. Healthy individuals who did not have cardiopulmonary, psychological, and neurological disorders or other impairments preventing execution of the research protocol and were not receiving treatment with medications that influence cardiac autonomic regulation. Systolic blood pressure (BP) < 140 mmHg or diastolic BP < 90 mmHg. Two subjects were excluded for presenting with BP > 140/90 mmHg.
Roque et al.^(23)^	21 individuals, female, age range of 18-30 years (25.2±3), from a similar socioeconomic background. Healthy individuals who did not have cardiopulmonary, psychological, and neurological disorders or other impairments preventing execution of the research protocol and were not receiving treatment with medications that influence cardiac autonomic regulation. Non-smoking individuals. Individuals with prior experience with musical instruments and classical ballet music and/or those who reported liking heavy metal and baroque musical styles were excluded from the studies. Individuals without hearing disorders.
Roque et al.^(19)^	40 individuals, female, age range of 18-35 years (25.9±4). Divided into two groups. Group 1: 21 individuals and Group 2: 19 individuals. Healthy individuals who did not have cardiopulmonary, psychological, and neurological disorders or other impairments preventing execution of the research protocol and were not receiving treatment with medications that influence cardiac autonomic regulation. Individuals with prior experience with musical instruments and classical ballet music and/or those who reported liking heavy metal and baroque musical styles were excluded from the studies. Individuals without hearing disorders.
Amaral et al.^(24)^	21 individuals, male, age range of 18-25 years (21.8±2). Healthy individuals who did not have cardiopulmonary, psychological, and neurological disorders or other impairments preventing execution of the research protocol and were not receiving treatment with medications that influence cardiac autonomic regulation. Non-smoking individuals. Individuals with prior experience with musical instruments and classical ballet music and/or those who reported liking heavy metal and baroque musical styles were excluded from the studies. Individuals without hearing disorders.
Amaral et al.^(25)^	16 individuals, male, age range of 18-25 years (20.7±3). Healthy individuals who did not have cardiopulmonary, psychological, and neurological disorders or other impairments preventing execution of the research protocol and were not receiving treatment with medications that influence cardiac autonomic regulation. Body mass index < 35 kg/m^2^. Non-smoking individuals. Individuals with prior experience with musical instruments and classical ballet music and/or those who reported liking heavy metal and baroque musical styles were excluded from the studies. Individuals without hearing disorders.
Silva et al.^(27)^	11 individuals, male, age range of 18-25 years (20.1±3). Healthy individuals who did not have cardiopulmonary, psychological, and neurological disorders or other impairments preventing execution of the research protocol and were not receiving treatment with medications that influence cardiac autonomic regulation. Individuals with prior experience with musical instruments and classical ballet music and/or those who reported liking heavy metal and baroque musical styles and individuals with prior experience with music therapy were excluded. Individuals without hearing disorders.
Silva and Guida^(28)^	12 healthy individuals, male, age range of 18-30 years (21.7±3). Healthy individuals who did not have cardiopulmonary, psychological, and neurological disorders or other impairments preventing execution of the research protocol and were not receiving treatment with medications that influence cardiac autonomic regulation. Individuals with prior experience with musical instruments and classical ballet music and/or those who reported liking heavy metal and baroque musical styles were excluded from the studies. Individuals without hearing disorders.
Amaral et al.^(26)^	28 healthy individuals, female, age range of 18-25 years (20.9±2.2). Healthy individuals who did not have cardiopulmonary, psychological, and neurological disorders or other impairments preventing execution of the research protocol and were not receiving treatment with medications that influence cardiac autonomic regulation. To prevent effects related to sexual hormones, women on days 11-15 and on days 21-25 after the first day of their menstrual cycle were not included. Individuals without hearing disorders.
Nogueira et al.^(29)^	22 individuals, female, age range of 18-30 years (20.8±2.7). Healthy individuals who did not have cardiopulmonary, psychological, and neurological disorders, without endocrine disorders, or other impairments preventing execution of the research protocol and were not receiving treatment with medications that influence cardiac autonomic regulation. Body mass index < 35 kg/m^2^. Systolic blood pressure (BP) < 140 mmHg or diastolic BP < 90 mmHg. To prevent effects related to sexual hormones, women on days 11-15 and on days 21-25 after the first day of their menstrual cycle were not included. Individuals without hearing disorders.

Caption: BP = Blood pressure

**Table 2:** Synthesis of the individual descriptive characteristics of the intervention, comparison, and outcomes of the included studies

**Author**	**Intervention, Comparison, and Outcomes**	
	**Auditory stimulation**	**HRV assessment**
Lee et al.^(30)^	Transducer: TDH-39 earphone, binaural presentation of the auditory stimuli. Type of auditory stimuli: white noise. Duration of auditory stimuli: 5 minutes. Intensity: 50, 60, 70, and 80 dB, presented in a random sequence, with 2-minutes intervals between intensities.	Equipment: ECG, amplifier, and analog converter (Model SS1C). Sampling rate: 256 Hz. Duration of HRV measurements in the presence of auditory stimuli: 5 minutes. Indices measured: Frequency domain: VLF (< 0.04 Hz), LF (0.04-0.15 Hz), HF (0.15-0.40 Hz), and LF/HF.
Roque et al.^(23)^	Transducer: insert earphones, binaural presentation of the auditory stimulus. Type of auditory stimulus: heavy metal and baroque musical styles. Duration of auditory stimulus: 4 minutes and 50 seconds and 5 minutes and 15 seconds. Intensity: approximately 70-80 dB, with 5-minute intervals between intensities.	Equipment: Polar RS800CX heart rate monitor. Sampling rate: 1000 Hz. At least 256 RR intervals were used for analysis and only series with more than 95% sinus beats were included. Duration of HRV measurements in the absence of auditory stimuli: 10 minutes. Duration of HRV measurements in the presence of auditory stimuli: 5 minutes. Indices measured: Time domain: SDNN, pNN50, RMSSD. Frequency domain: LF (0.04-0.15 Hz), HF (0.15-0.40 Hz), and LF/HF.
Roque et al.^(19)^	Transducer: insert earphones, binaural presentation of the auditory stimulus. Type of auditory stimulus: heavy metal, baroque musical styles and white noise. Duration of auditory stimulus: 4 minutes and 50 seconds, 5 minutes and 5 minutes and 15 seconds. Intensity: approximately 70-80 dB and 90 dB, with 5-minute intervals between intensities.	Equipment: Polar RS800CX heart rate monitor. Sampling rate: 1000 Hz. At least 256 RR intervals were used for analysis and only series with more than 95% sinus beats were included. Duration of HRV measurements in the absence of auditory stimuli: 10 minutes. Indices measured: Groups 1 and 2 – Geometric analysis, Group 2 – Time domain and frequency domain. Time domain: SDNN, pNN50, RMSSD. Frequency domain: LF (0.04-0.15 Hz), HF (0.15-0.40 Hz), and LF/HF. Geometric analysis: RRTri, TINN, SD1, SD2, and SD1/SD2.
Amaral et al.^(24)^	Transducer: insert earphones, binaural presentation of the auditory stimulus. Type of auditory stimulus: heavy metal and baroque musical styles. Duration of auditory stimulus: 4 minutes and 50 seconds and 5 minutes and 15 seconds. Intensity: blocks of 60-70 dB, 70-80 dB, and 80-90 dB, with 5-minute intervals between intensities.	Equipment: Polar RS800CX heart rate monitor. Sampling rate: 1000 Hz. At least 256 RR intervals were used for analysis and only series with more than 95% sinus beats were included. Duration of HRV measurements in the absence of auditory stimuli: 10 minutes. Duration of HRV measurements in the presence of auditory stimuli: 4 minutes and 50 seconds. Indices measured: Time domain: SDNN, pNN50, RMSSD. Frequency domain: LF (0.04-0.15 Hz), HF (0.15-0.40 Hz), and LF/HF.
Amaral et al.^(25)^	Transducer: insert earphones, binaural presentation of the auditory stimulus. Type of auditory stimulus: heavy metal and baroque musical styles. Duration of auditory stimulus: 4 minutes and 50 seconds and 5 minutes and 15 seconds. Intensity: blocks of 60-70 dB, 70-80 dB, and 80-90 dB, with 5-minute intervals between intensities.	Equipment: Polar RS800CX heart rate monitor. Sampling rate: 1000 Hz. At least 256 RR intervals were used for analysis and only series with more than 95% sinus beats were included. Duration of HRV measurements in the absence of auditory stimuli: 10 minutes. Duration of HRV measurements in the presence of auditory stimuli: 4 minutes and 50 seconds and 5 minutes. Indices measured: Geometric analysis: RRTri, TINN, SD1, SD2, and SD1/SD2.
Silva et al.^(27)^	Transducer: insert earphones, binaural presentation of the auditory stimulus. Type of auditory stimulus: heavy metal and baroque musical styles. Duration of auditory stimulus: 4 minutes and 50 seconds and 5 minutes and 15 seconds. Intensity: 64-85 dB, with 5-minute intervals between intensities.	Equipment: Polar RS800CX heart rate monitor. Sampling rate: 1000 Hz. At least 256 RR intervals were used for analysis and only series with more than 95% sinus beats were included. Duration of HRV measurements in the absence of auditory stimuli: 10 minutes. Duration of HRV measurements in the presence of auditory stimuli: 5 minutes. Indices measured: Geometric analysis: RRTri, TINN, SD1, SD2, and SD1/SD2.
Silva and Guida^(28)^	Transducer: insert earphones, binaural presentation of the auditory stimulus. Type of auditory stimulus: heavy metal and baroque musical styles. Duration of auditory stimulus: 4 minutes and 50 seconds and 5 minutes and 15 seconds. Intensity: 64-85 dB, with 5-minute intervals between intensities.	Equipment: Polar RS800CX heart rate monitor. Sampling rate: 1000 Hz. At least 256 RR intervals were used for analysis and only series with more than 95% sinus beats were included. Duration of HRV measurements in the absence of auditory stimuli: 10 minutes. Duration of HRV measurements in the presence of auditory stimuli: 5 minutes. Indices measured: Geometric analysis: RRTri, TINN, SD1, SD2, and SD1/SD2.
Silva and Guida^(28)^	Transducer: insert earphones, binaural presentation of the auditory stimulus. Type of auditory stimulus: heavy metal and baroque musical styles. Duration of auditory stimulus: 4 minutes and 50 seconds and 5 minutes and 15 seconds. Intensity: 64-85 dB, with 5-minute intervals between intensities.	Equipment: Polar RS800CX heart rate monitor. Sampling rate: 1000 Hz. At least 256 RR intervals were used for analysis and only series with more than 95% sinus beats were included. Duration of HRV measurements in the absence of auditory stimuli: 10 minutes. Duration of HRV measurements in the presence of auditory stimuli: 5 minutes. Indices measured: Geometric analysis: RRTri, TINN, SD1, SD2, and SD1/SD2.
Amaral et al.^(26)^	Transducer: insert earphones, binaural presentation of the auditory stimulus. Type of auditory stimulus: heavy metal and baroque musical styles. Duration of the auditory stimulus: unspecified. Intensity: blocks of 60-70 dB, 70-80 dB, and 80-90 dB.	Equipment: Polar RS800CX heart rate monitor. Sampling rate: 1000 Hz. At least 256 RR intervals were used for analysis and only series with more than 95% sinus beats were included. Duration of HRV measurements in the absence of auditory stimuli: 10 minutes. Duration of HRV measurements in the presence of the auditory stimulus: 10 minutes. Indices measured: Time domain: SDNN, pNN50, RMSSD. Frequency domain: LF (0.04-0.15 Hz), HF (0.15-0.40 Hz), and LF/HF.
Nogueira et al.^(29)^	Transducer: insert earphones, binaural presentation of the auditory stimuli. Type of auditory stimuli: heavy metal musical style. Duration of the auditory stimuli: 5 minutes and 15 seconds. Intensity: 75-84 dB, approximately.	Equipment: Polar RS800CX heart rate monitor. Sampling rate: 1000 Hz. At least 1000 RR intervals were used for analysis and only series with more than 95% sinus beats were included. Duration of HRV measurements in the absence and presence of the auditory stimulus: 20 minutes each. Indices measured: Time domain: SDNN/RMSSD. Frequency domain: LF (0.04-0.15 Hz), HF (0.15-0.40 Hz), and LF/HF. Geometric analysis: RRTri, TINN, SD1, SD2, and SD1/SD2.

### Results of the Individual Studies

The studies by Lee et al.^(30)^, LF (ms^2^) and HF (ms^2^) indices; Roque et al.^(23)^, LF(ms^2^) index; Roque et al.^(19)^, RRTri, SD2 and HF (n.u.) indices; Silva and Guida^(28)^, RRTri and SD2 indices; and Amaral et al.^(26)^, SDNN and LF(ms^2^) indices, verified significant differences for the various HRV indices, in silence and in the presence of the acoustic stimulation, in healthy individuals with normal hearing.

On the other hand, in the studies by Amaral et al.^(24,25)^, Silva et al.^(27)^, and Nogueira et al.^(29)^ no were found effects of acoustic stimulation on the HRV.

Table 3 synthesizes the characteristics of the outcomes of the included studies using p-values.

**Table 3:** Synthesis of the characteristics of the outcomes of the included studies, by p-values

**Author**	**Outcomes (p-values)**
Lee et al.^(30)^	White noise: frequency domain – LF(ms^2^): p < 0.01*. Only the control was significantly lower than white noise at intensities between 50–80 dB (p < 0.05)*. HF(ms^2^): p = 0.74 and LHR: p < 0.01*.
Roque et al.^(23)^	Baroque and heavy metal: time domain – SDNN: p = 0.12, RMSSD: p = 0.8, pNN50: p = 0.9. Frequency domain – LF(ms^2^): p = 0.025*(heavy metal), HF(ms^2^): p = 0.1, LF(n.u.): p = 0.8, HF(n.u.): p = 0.8, LF/HF: p = 0.7.
Roque et al.^(19)^	Group 1: Baroque and heavy metal: geometric analysis – RRTri: p = 0.03*, TINN: p = 0.2. Poincaré plot – SD1: p = 0.09, SD2: p = 0.04*, SD1/SD2: p = 0.56. Group 2: baroque, heavy metal, and white noise: geometric analysis – RRTri: p = 0.1, TINN: p = 0.1. Poincaré plot – SD1: p = 0.5, SD2: p = 0.09, SD1/SD2: p = 0.39. Time domain – SDNN: p = 0.37, RMSSD: p = 0.3, pNN50: p = 0.17. Frequency domain – LF(ms^2^): p = 0.12, LF(n.u.): p = 0.2, HF(ms^2^): p = 0.19, HF(n.u.): p = 0.04*(white Noise), LF/HF: p = 0.08.
Amaral et al.^(24)^	Time domain: (i) heavy metal – RMSSD: p = 0.5, pNN50: p = 0.4, SDNN: p = 0.47. (ii) Baroque – RMSSD: p = 0.7, pNN50: p = 0.6, SDNN: p = 0.6. Frequency domain: (i) heavy metal – HF(ms^2^): p = 0.5, LF(ms^2^): p = 0.6, HF(n.u.): p = 0.45, LF(n.u.): p = 0.67, LF/HF: p = 0.5. (ii) baroque – HF(ms^2^): p = 0.56, LF(ms^2^): p = 0.77, HF(n.u.): p = 0.45, LF(n.u.): p = 0.54, LF/HF: p = 0.58.
Amaral et al.^(25)^	Geometric analysis: (i) heavy metal – RRTri: p = 0.4, TINN: p = 0.6. Poincaré plot – SD1: p = 0.4, SD2: p = 0.4, SD1/SD2: p = 0.5. (ii) baroque – RRTri: p = 0.3, TINN: p = 0.5. Poincaré plot – SD1: p = 0.5, SD2: p = 0.6, SD1/SD2: p = 0.4.
Silva et al.^(27)^	Time domain: (i) heavy Metal – RMSSD: p = 0.8, pNN50: p = 0.8, SDNN: p = 0.7. (ii) baroque – RMSSD: p = 0.75, pNN50: p = 0.67, SDNN: p = 0.76. Frequency domain: (i) heavy metal – HF(ms^2^): p = 0.64, LF(ms^2^): p = 0.71, HF(n.u.): p = 0.79, LF(n.u.): p = 0.68, LF/HF: p = 0.9. (ii) baroque – HF(ms^2^): p = 0.8, LF(ms^2^): p = 0.5, HF(n.u.): p = 0.76, LF(n.u.): p = 0.7, LF/HF: p = 0.82.
Silva and Guida^(28)^	Baroque and heavy metal: geometric analysis – RRTri: p = 0.04*(heavy metal), TINN: p = 0.07. Poincaré plot – SD1: p = 0.09, SD2: p = 0.03*(heavy metal), SD1/SD2: p = 0.076.
Amaral et al.^(26)^	Time domain: (i) heavy metal – RMSSD: p = 0.97, pNN50: p = 0.98, SDNN: p = 0.01*(80-90dB). (ii) baroque – RMSSD: p = 0.65, pNN50: p = 0.89, SDNN: p = 0.34. Frequency domain: (i) heavy metal – HF(ms^2^): p = 0.11, LF(ms^2^): p = 0.04*(60-70dB), HF(n.u.): p = 0.82, LF(n.u.): p = 0.83, LF/HF: p = 0.86. (ii) baroque – HF(ms^2^): p = 0.73, LF(ms^2^): p = 0.03*(60–70dB), HF(n.u.): p = 1.00, LF(n.u.): p = 0.99, LF/HF: p = 0.95.
Nogueira et al.^(29)^	Heavy metal: Time domain – SDNN: p = 0.11, RMSSD: p = 0.1, SDNN/RMSSD: p = 0.34, pNN50: p = 0.12. Frequency domain – LF(ms^2^): p = 0.06, LF(n.u.): p = 0.071, HF(ms^2^): p = 0.26, HF(n.u.): p = 0.07, LF/HF: p = 0.05*. Geometric analysis – RRTri: p = 0.11, TINN: p = 0.077. Poincaré plot – SD1: p = 0.1, SD2: p = 0.1, SD1/SD2: p = 0.45.

* Statistically significant differences

### Risk of Bias Assessment

**Figure 2:**
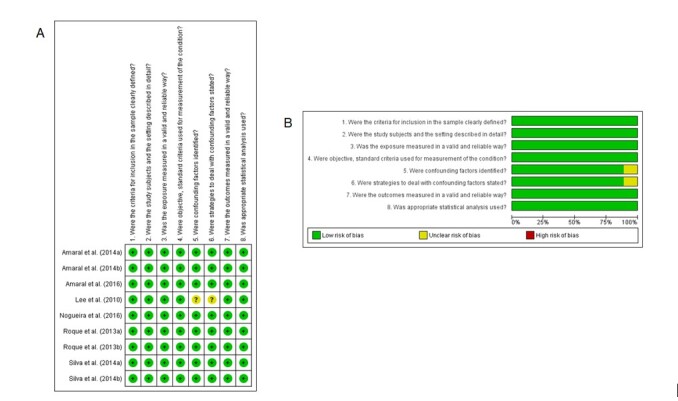
Summary of risk of bias assessed by Joanna Briggs Institute Critical Appraisal Checklist for Analytical Cross-Sectional Studies, with author’s judgments for each study included

The studies were assessed using the JBI Critical Appraisal Checklist for Analytical Cross Sectional Studies. All studies included in this review were classified as having a “low risk” of bias. The Figure 2 summarize the evaluations obtained by the JBI tool.

Caption: + = Low risk of bias, ? = Unclear risk of bias. Source: Software Review Manager version 5.4. (Cochrane Collaboration)

Source: Software Review Manager version 5.4. (Cochrane collaboration)

### Synthesis of Results - meta-analysis

#### Primary Outcomes

Data for the primary outcomes were presented as forest plot graphs, with the mean differences estimated for the combined studies.

**Figure 3:**
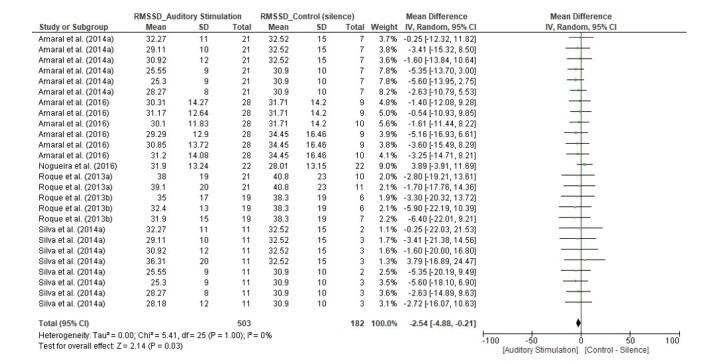
Forest Plot for the RMSSD index

Source: Software Review Manager version 5.4. (Cochrane collaboration)

**Figure 4:**
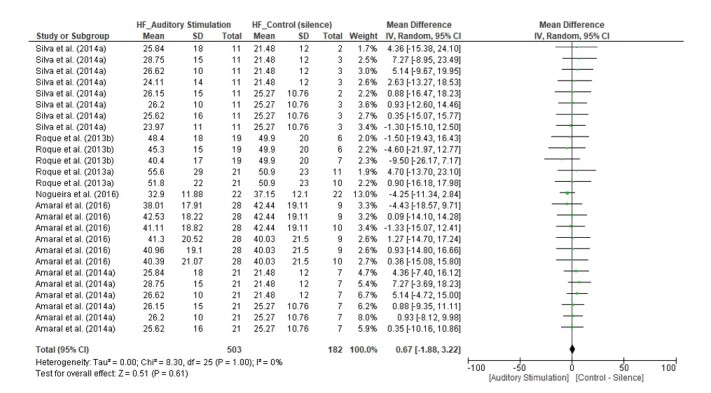
Forest Plot for the HF(n.u.) index

Source: Software Review Manager version 5.4. (Cochrane Collaboration)

**Figure 5:**
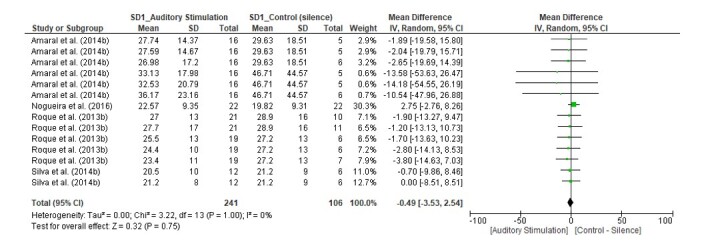
Forest Plot for the SD1 index

Source: Software Review Manager version 5.4. (Cochrane collaboration)

We found a statistically significant difference in favor of the auditory stimulus only for the RMSSD index (MD = -2.54, 95% CI [-4.88, -0.21], Z = 2.14, p = 0.03), with I^2^ = 0% (Figure 3). The HF(n.u.) index (MD = 0.67, 95% CI [-1.88, 3.22], Z = 0.51, p = 0.61) with I^2^ = 0%, and the SD1 index (MD = -0.49, 95% CI [-3.53, 2.54], Z = 0.32, p = 0.75) with I^2^ = 0% did not present significant differences (Figures 4 and 5, respectively).

### Certainty Assessment (primary outcomes)

Since all studies included were cross-sectional, the certainty of evidence was reduced, with low evidence (Table 4).

**Table 4:** GRADE evidence profile for the primary outcomes

**Certainty assessment**								**Effect**		**Certainty**
**Outcomes**	**Nº of studies**	**Study design**	**Risk of bias**	**Inconsistency**	**Indirectness**	**Imprecision**	**Other considerations**	**Relative** **(95% CI)**	**Absolute** **(95% CI)**	
RMSSD	6	observational studies	not serious	not serious	not serious	not serious	none	-	MD **2.54 lower** (4.88 lower to 0.21 lower)	⨁⨁◯◯ Low
HF(n.u)	6	observational studies	not serious	not serious	not serious	not serious	none	-	MD **0.67 higher** (1.88 lower to 3.22 higher)	⨁⨁◯◯ Low
SD1	4	observational studies	not serious	not serious	not serious	not serious	none	-	MD **0.49 lower** (3.53 lower to 2.54 higher)	⨁⨁◯◯ Low

Caption: CI = Confidence interval; MD = Mean difference

Source: GRADEpro (GDT) (©2021) Source: GRADEpro GDT^(22)^

#### Secondary Outcomes

Statistically significant differences were found for the pNN50 index (MD = -2.33, 95% CI [-4.07, -0.59], Z = 2.62, p = 0.009) with I^2^ = 0%; the SDNN index (MD = -5.88, 95% CI [-8.26, -3.49], Z = 4.83, p <0.00001) with I^2^ = 0%; the RRTri index (MD = -1.20, 95% CI [-2.23, -0.17], Z = 2.29, p = 0.02) with I^2^ = 0%; and the SD2 index (MD = -5.33, 95% CI [-10.70, 0.04], Z = 1.95, p = 0.05), with I^2^ = 0%. In contrast, no statistically significant differences were observed for the HF(ms^2^) index (MD = -55.28, 95% CI [-146.27, 35.70], Z = 1.19, p = 0.23) with I^2^ = 58%; the LF(ms^2^) index (MD = 132.10, 95% CI [-23.62, 287.82], Z = 1.66, p = 0.10) with I^2^ = 62%; the LF(n.u.) index (MD = -0.99, 95% CI [-3.59, 1.60], Z = 0.75, p = 0.45) with I^2^ = 0%; the LF/HF index (MD = -0.1, 95% CI [-0.31, 0.29], Z = 0.05, p = 0.96) with I^2^ = 0%; the TINN index (MD = -7.15, 95% CI [-25.29, 10.98], Z = 0.77, p = 0.44) with I^2^ = 0%; and the SD1/SD2 index (MD = -0.02, 95% CI [-0.06, 0.01], Z = 1.51, p = 0.13), with I^2^ = 0%. The evidence for all the indices of the secondary outcomes had a low classification.

Based on the results of the meta-analysis, the RMSSD, pNN50, SDNN, RRTri, and SD2 indices exhibited significant differences in the presence of auditory stimuli, while the HF(ms^2^), HF(n.u.), LF(ms^2^), LF(n.u.), LF/HF, TINN, SD1, and SD1/SD2 indices did not change (p-values). It is important to highlight the power of meta-analysis, because when analyzing, for example, the effect size of six cross-sectional studies that evaluated the RMSSD index of HRV, none of the studies showed statistically significant effects, although when the data from all of the studies weew groped together, the sample size increased and, consequently, the statistical power improved, demonstrating the effects of acoustic stimulation on the RMSSD index. However, the results obtained (primary and secondary outcomes) must be interpreted with caution when considering the effect size of clinical outcomes close to the vertical line of the null hypothesis^(31)^.

In this systematic review, we did not include studies on individuals with hearing loss or studies on children, regardless of hearing level. The studies on these populations were not eligible due to exclusion criteria related to the intervention^(1,2,7,8,32,33)^. As a result, the analysis was performed only in healthy adults without hearing loss.

### Primary Outcomes

The absence of statistical heterogeneity across the studies indicates that potential clinical and methodological differences did not influence the results, which substantiates the degree of confidence obtained.

In our analysis of the data groups, we found a significant difference only for the RMSSD index (Figure 3), with reduced parasympathetic activity in healthy adults with normal hearing. On the other hand, we did not observe significant differences for the HF(n.u.) and SD1 indices (Figures 4 and 5), although these indices are similarly related to the analysis of parasympathetic behavior.

These findings suggest that the RMSSD index may demonstrate better accuracy in the presence of auditory stimuli. It is important to analyze the variability contained in the analysis of the HRV indices. For example, for healthy adults aged 20-40 years, the RMSSD index with a mean of 53.1 and a standard deviation of ±22.2 can be considered^(34)^, which influences the CI obtained. The wider CI increases the imprecision and consequently the uncertainty about the effect of the evidence.

### Secondary Outcomes

The pNN50, SDNN, RRTri, and SD2 indices exhibited significant differences, evidencing a reduction in the general and vagal autonomic modulation of the heart upon auditory stimulation. Conversely, we found no effects on the HF(ms^2^), LF(ms^2^), LF(n.u.), LF/HF, TINN, and SD1/SD2 indices.

Only one study included in the meta-analysis used white noise as a stimulus, which precluded its comparability in isolation^(19)^; nevertheless, the individual results of the study revealed a reduction in parasympathetic activation and a greater propensity for sympathetic activation, evidenced by the LF/HF ratio, corroborating findings by Lee et al.^(30)^, which are included in the descriptive synthesis.

Therefore, the RMSSD, pNN50, SDNN, RRTri, and SD2 indices demonstrated a relationship between hearing and RR interval variability, pointing to their potential use for hearing purposes. However, interpretations of these findings should be made with reservations as the HF(ms^2^), HF(n.u.), LF(ms^2^), LF(n.u.), LF/HF, TINN, SD1, and SD1/SD2 indices did not present significant differences in the presence of auditory stimuli.

Accordingly, we encourage discussions about the specific conditions needed for auditory stimulation to effect control over heart rate, which could explain the results found in the individual studies^(19,23,24,26,27,29)^. Moreover, the low quality of the evidence included in this review, which is due to the fact that all the studies were cross-sectional observational studies with a low effect size, further indicate that caution is needed in interpreting the findings. It also highlights the limitations of this systematic review.

We recommend further research in this direction to increase the quality of evidence and elucidate existing questions—for instance, sensitivity to other auditory stimuli, such as a click or a pure tone, in audiological evaluations. This is because the spectral characteristics of auditory stimuli can influence the results obtained. Additionally, it is important to investigate the generalization of these findings in children, given the specificity of age in analyses of the autonomic nervous system, as well as the application of these findings in individuals with hearing loss. Thus, more evidence is needed to consider the use of HRV as an alternative for hearing screening.

## CONCLUSION

In conclusion, it is suggested auditory stimulation may influence the RMSSD, pNN50, SDNN, RRTri, and SD2 indices of HRV in healthy adults with normal hearing (p-values). The results of the meta-analysis should be interpreted with caution when considering the effect size of primary and secondary outcomes close to the null line. We emphasize the importance of future studies in the area.

## Appendix A Database search strategy

**Table:**

**Database**	**Search (November 15, 2021; updated on November 10, 2022)**	
**Cochrane Library**		
(“Acoustic Stimulation” OR “Auditory Stimulation”) AND (“Heart Rate” OR “Heart Rates” OR “Heart Rate Determination” OR “Autonomic Nervous System” OR “Autonomic Nervous Systems” OR “Parasympathetic Nervous System” OR “Parasympathetic Nervous Systems” OR “Sympathetic Nervous System” OR “Sympathetic Nervous Systems” OR “Vagus Nerve” OR “Cranial Nerve X” OR “Heart Rate Variability” OR “Cardiac Period”) AND (“Hearing” OR “Audition” OR “Hearing Loss” OR “Hypoacusis” OR “Deafness”) in Title Abstract Keyword		
**Embase**		
('acoustic stimulation':ti,ab,kw OR 'auditory stimulation':ti,ab,kw) AND ('heart rate':ti,ab,kw OR 'heart rates':ti,ab,kw OR 'heart rate determination':ti,ab,kw OR 'autonomic nervous system':ti,ab,kw OR 'autonomic nervous systems':ti,ab,kw OR 'parasympathetic nervous system':ti,ab,kw OR 'parasympathetic nervous systems':ti,ab,kw OR 'sympathetic nervous system':ti,ab,kw OR 'sympathetic nervous systems':ti,ab,kw OR 'vagus nerve':ti,ab,kw OR 'cranial nerve x':ti,ab,kw OR 'heart rate variability':ti,ab,kw OR 'cardiac period':ti,ab,kw) AND ('hearing':ti,ab,kw OR 'audition':ti,ab,kw OR 'hearing loss':ti,ab,kw OR 'hypoacusis':ti,ab,kw OR 'deafness':ti,ab,kw)		
**LILACS**		
#1Title, abstract, subject: ((“Acoustic Stimulation” OR “Auditory Stimulation”)) AND ((“Heart Rate” OR “Heart Rates” OR “Heart Rate Determination” OR “Autonomic Nervous System” OR “Autonomic Nervous Systems” OR “Parasympathetic Nervous System” OR “Parasympathetic Nervous Systems” OR “Sympathetic Nervous System” OR “Sympathetic Nervous Systems” OR “Vagus Nerve” OR “Cranial Nerve X” OR “Heart Rate Variability” OR “Cardiac Period”)) AND ((“Hearing” OR “Audition” OR “Hearing Loss” OR “Hypoacusis” OR “Deafness”))#2Título, resumen, asunto: ((“Estimulación Acústica”) AND ((“Frecuencia Cardíaca” OR “Determinación de la Frecuencia Cardíaca” OR “Sistema Nervioso Autónomo” OR “Sistema Nervioso Parasimpático” OR “Sistema Nervioso Simpático” OR “Nervio Vago” OR “Variabilidad del Ritmo Cardíaco” OR “Período Cardíaco”)) AND ((“Audición” OR “Pérdida Auditiva” OR “Sordera”)		
**PubMed/Medline**		
(((“Acoustic Stimulation” OR “Auditory Stimulation”)) AND ((“Heart Rate” OR “Heart Rates” OR “Heart Rate Determination” OR “Autonomic Nervous System” OR “Autonomic Nervous Systems” OR “Parasympathetic Nervous System” OR “Parasympathetic Nervous Systems” OR “Sympathetic Nervous System” OR “Sympathetic Nervous Systems” OR “Vagus Nerve” OR “Cranial Nerve X” OR “Heart Rate Variability” OR “Cardiac Period”))) AND ((“Hearing” OR “Audition” OR “Hearing Loss” OR “Hypoacusis” OR “Deafness”))		
**Scopus**		
TITLE-ABS-KEY ((“Acoustic Stimulation” OR “Auditory Stimulation”)) AND ((“Heart Rate” OR “Heart Rates” OR “Heart Rate Determination” OR “Autonomic Nervous System” OR “Autonomic Nervous Systems” OR “Parasympathetic Nervous System” OR “Parasympathetic Nervous Systems” OR “Sympathetic Nervous System” OR “Sympathetic Nervous Systems” OR “Vagus Nerve” OR “Cranial Nerve X” OR “Heart Rate Variability” OR “Cardiac Period”)) AND ((“Hearing” OR “Audition” OR “Hearing Loss” OR “Hypoacusis” OR “Deafness”))		
**Web of Science**		
TS=(“Acoustic Stimulation” OR “Auditory Stimulation”) AND TS=(“Heart Rate” OR “Heart Rates OR “Heart Rate Determination” OR “Autonomic Nervous System” OR “Autonomic Nervous Systems” OR “Parasympathetic Nervous System” OR “Parasympathetic Nervous Systems” OR “Sympathetic Nervous System” OR “Sympathetic Nervous Systems” OR “Vagus Nerve” OR “Cranial Nerve X” OR “Heart Rate Variability” OR “Cardiac Period”) AND TS=(“Hearing” OR “Audition” OR “Hearing Loss” OR “Hypoacusis” OR “Deafness”)		
**Google Scholar**		
(“Acoustic Stimulation”) AND (“Heart Rate” OR “Autonomic Nervous System” OR “Heart Rate Variability”) AND (“Hearing” OR “Hearing Loss”) filetype:pdf		
**Opengrey**		
#1(“Acoustic Stimulation” OR “Auditory Stimulation”) AND (“Heart Rate” OR “Heart Rates” OR “Heart Rate Determination” OR “Autonomic Nervous System” OR “Autonomic Nervous Systems” OR “Parasympathetic Nervous System” OR “Parasympathetic Nervous Systems” OR “Sympathetic Nervous System” OR “Sympathetic Nervous Systems” OR “Vagus Nerve” OR “Cranial Nerve X” OR “Heart Rate Variability” OR “Cardiac Period”) AND (“Hearing” OR “Audition” OR “Hearing Loss” OR “Hypoacusis” OR “Deafness”)		
**ProQuest (Dissertation and Thesis)**		
(“Acoustic Stimulation” OR “Auditory Stimulation”) AND (“Heart Rate” OR “Heart Rates” OR “Heart Rate Determination” OR “Autonomic Nervous System” OR “Autonomic Nervous Systems” OR “Parasympathetic Nervous System” OR “Parasympathetic Nervous Systems” OR “Sympathetic Nervous System” OR “Sympathetic Nervous Systems” OR “Vagus Nerve” OR “Cranial Nerve X” OR “Heart Rate Variability” OR “Cardiac Period”) AND (“Hearing” OR “Audition” OR “Hearing Loss” OR “Hypoacusis” OR “Deafness”)		

## Appendix B Excluded articles and reasons for exclusion (n = 47)

**Table:**

**Article**	**Reason for exclusion**	
Weihs et al. (1954)	1	
Neuberger and Schmid (1960)	1	
Brackbill et al. (1966)	1	
Raskin et al. (1969)	1	
Hord and Ackerland (1971)	1	
Lewis (1971)	1	
Jeffrey and Cohen (1971)	1	
Berg (1972)	1	
Delfini and Campos (1972)	1	
Gautier (1972)	1	
Turkewitz et al. (1972a)	1	
Turkewitz et al. (1972b)	1	
Campos and Brackbill (1973)	1	
Chüden (1973)	1	
Stratton and Connolly (1973)	1	
Kearsley (1973)	1	
Schulman (1973)	1	
Brzezinska et al. (1974)	1	
Schulman (1974)	1	
Kinney and Kagan (1976)	1	
Suzuki (1978)	1	
Kobayashi (1978)	1	
Borton and Smith (1980)	1	
Brackbill et al. (1982)	1	
Johansson et al. (1982)	1	
Morrongiello et al. (1982)	1	
Rossi et al. (1982)	1	
Millot et al. (1987)	1	
Fernández and Vila (1989)	1	
Iwanaga and Tsukamoto (1997)	1	
Wharrad and Davis (1997)	1	
Rozhkov and Anurova (2000)	1	
Guilleminault et al. (2006)	1	
Kirillova et al. (2007)	1	
Salimpoor et al. (2009)	1	
Roy et al. (2012)		1
Castro et al. (2013)		1
Mastnak (2014)		1
Jäncke et al. (2015)		1
Mackersie et al. (2015)		1
Chuen et al. (2016)		1
Trappe and Voit (2016)		1
Lynar et al. (2017)		2
Mackersie and Kearney (2017)		1
Mojtabavi et al. (2021)		1
Bakaeva et al. (2022)		1
Ubrangala et al. (2022)		1

Caption: Reason 1 = exclusion due to intervention; Reason 2 = exclusion due to outcome
